# Physical fitness and body composition assessments in advanced cancer patients undergoing exenterative surgery – A pilot cohort study

**DOI:** 10.1111/codi.70298

**Published:** 2025-11-18

**Authors:** Marina Looby, Lewis Matthews, Charles T. West, Kashuf Khan, Gillian Ansell, Kathryn Donovan, Laura Wood, Patrick Tapley, Rhys Lewis, Kate Stoddard, Michael P. W. Grocott, Sandy Jack, Hideaki Yano, Denny Levett, Alex Mirnezami, Malcolm A. West

**Affiliations:** ^1^ Academic Surgery, Cancer Sciences, Faculty of Medicine University of Southampton Southampton UK; ^2^ Southampton Complex Cancer and Exenteration Team (SCCET) University Hospital Southampton Southampton UK; ^3^ NIHR Biomedical Research Centre, Perioperative Medicine and Prehabilitation Theme University Hospital Southampton Southampton UK; ^4^ Department of Anaesthesia and Perioperative Medicine University Hospital Southampton Southampton UK

**Keywords:** cardiopulmonary exercise testing, exenteration surgery, myosteatosis, physical fitness, sarcopenia

## Abstract

**Aim:**

Locally advanced pelvic malignancies, such as colorectal and anal cancers, can only be cured through multimodal cancer treatment including multi‐visceral exenterative resections, which carry a high mortality and morbidity risk. Despite strong predictive abilities in other cancer cohorts, the combined prognostic value of body composition and cardiopulmonary exercise testing (CPET) for major in‐hospital morbidity in patients undergoing exenterative surgery for advanced pelvic cancers has not been evaluated.

**Method:**

A locally advanced colorectal and anal cancer cohort was derived from a prospectively maintained quaternary database. CPET was undertaken preoperatively, according to national guidelines. Skeletal muscle index (SMI) and radiation attenuation (SM‐RA) were obtained from analysing L3 slices from preoperative computed tomography scans using SliceOmatic 5.0 and classified using predefined thresholds. Major morbidity was defined as Clavien‐Dindo classification 3a or greater.

**Results:**

From 247 patients (58% male, median age 60 years), 62.4% and 35.5% had locally advanced or recurrent disease respectively. Physical fitness variables were significantly reduced in low SMI or low SM‐RA patients. In multivariate linear regression, SMI was strongly predictive of oxygen uptake at the anaerobic threshold (B = 0.013, *p* = 0.001) and at peak (B = 0.015, *p* = 0.002). 17.3% of all patients experienced a major postoperative complication. In multivariate analysis, reduced peak power output (<1.5 W kg^−1^) was significantly associated with an increased risk of postoperative major morbidity (OR = 2.6, *p* = 0.012).

**Conclusion:**

CPET may be predictive of in‐hospital major morbidity in this cohort. The association of CPET with body composition necessitates further evaluation and external validation in a larger patient cohort, specifically interrogating their combined role in morbidity prediction and as a target for prehabilitation interventions.

**What does this paper add to the literature?**

This study evaluates the combined prognostic value of body composition and cardiopulmonary exercise testing (CPET) for in‐hospital major morbidity post‐exenterative surgery for advanced pelvic cancers, finding that only selected CPET variables are linked to major morbidity, despite strong associations between body composition and CPET variables.

## INTRODUCTION

Exenterative surgery, specifically pelvic exenteration, is a technically challenging and bespoke multi‐visceral resection. Exenteration refers to radical surgery to remove advanced or recurrent pelvic, and occasionally abdominal malignancies by partial or total resection of contiguously affected organs and/or structures [1]. *En bloc* cancer surgery aims to obtain negative cancer resection margins (R0) in locally advanced and locally recurrent colorectal and anal cancers, improving long‐term prognosis [1, 2]. Despite careful patient selection, exenterative surgery carries a high in‐hospital morbidity risk, with major complication rates of up to 87% [3], with up to 33% of patients requiring further interventions [4]. Neoadjuvant treatments (NAT), often used before exenterative surgery can improve oncological outcomes [5], but may increase major postoperative complication risk due to associated toxicities and reductions in whole‐body muscle mass and physical fitness [5, 6]. Given the dual challenges posed by NAT and exenterative surgery, identifying modifiable factors to recognise and potentially optimise high‐risk patients preoperatively is an urgent unmet need.

**FIGURE 1:**
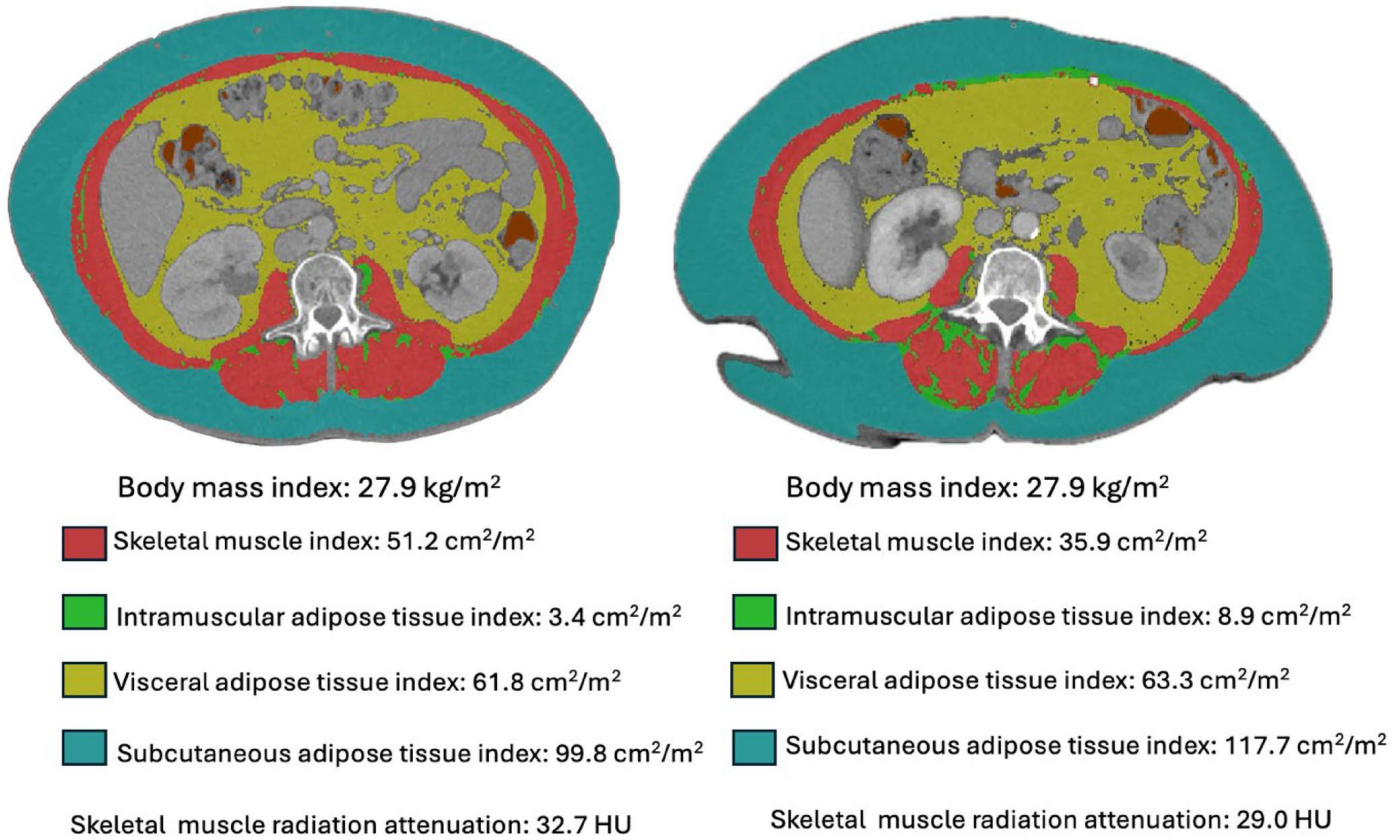
SliceOmatic analysis at L3 showing differing body compositions for the same body mass index.

Sarcopenia, defined as the progressive and generalised loss of skeletal muscle mass and strength [7], and myosteatosis, defined as adipose infiltration of skeletal muscle tissue, are measures of body composition associated with ageing, malnutrition and cachexia and therefore are key indicators of physical resilience in cancer patients. Both have shown strong associations with adverse postoperative outcomes in colorectal cancer patients [8, 9, 10], especially in locally advanced disease following NAT [11]. However, research specific to exenterative surgery for advanced and recurrent cancers is limited and requires further investigation. To date a single small cohort study reported no association between sarcopenia and postoperative morbidity risk [12]. Individuals with the same BMI may have contrasting body compositions (Figure 1) and tools to evaluate nutritional status such as body mass index (BMI) or the Malnutrition Universal Screening Tool [13] fail to account for differences in fat and muscle proportions, as well as the effects of age, sex, and ethnicity. Body composition is more precisely assessed through computed tomography (CT) scan single‐slices at the third lumbar vertebra (L3), using measures such as skeletal muscle index (SMI), a measure of muscle quantity, and skeletal muscle radiation attenuation (SM‐RA), a measure of muscle quality, which have strong correlation to MRI‐derived and whole‐body derived body composition values [14].

Aerobic exercise capacity (physical fitness), measured using cardiopulmonary exercise testing (CPET), is the gold standard for the objective assessment of fitness and may be used to predict perioperative risk. Accurate and reproducible CPET variables such as oxygen uptake at peak (VO_2_ peak) and at anaerobic threshold (VO_2_ AT), with value ranges of 16.7–18.0 mL kg^−1^ min^−1^ and 10.1–11.1 mL kg^−1^ min^−1^ respectively have been predictive of postoperative complications in colorectal cancer surgery [15]. These variables have not been evaluated as prognostic factors specifically prior to exenterative surgery. Additionally, peak power output (PPO), which is the power output in watts at VO_2_ peak, has been reported to be associated with risk in major abdominal surgery [16], and may be beneficial to evaluate as a prognostic factor before exenterative surgery. Moreover, CPET provides opportunity for identifying and correcting factors leading to exercise intolerance, for example, cardiorespiratory factors or anaemia, as well as prescribing exercise prehabilitation, demonstrated to improve postoperative outcomes [17, 18].

Advantageous body compositions, with better muscle quantity and quality, are assumed to be associated with increased physical fitness and vice versa. However, the interplay between these factors has only recently been studied, revealing in multivariate analysis that low SMI is negatively associated with VO_2_ AT [19] and with VO_2_ peak [10]. Evaluating body composition and physical fitness together in the context of exenterative surgery may determine their predictive value in conjunction and isolation. CPET is currently still costly; it is dependent on specialist staff/equipment and is time‐consuming, whereas body composition assessment using routine cancer staging CT images is readily accessible and less costly than CPET. However, body composition assessment still requires specialist software, human image manipulation, and specific CT contrast phases and sequences to afford generalisability.

In this study, the primary aim was to assess preoperative abdominal body composition and physical fitness in a locally advanced and recurrent cancer pilot cohort prior to exenterative surgery. The secondary aims were to interrogate (A) the relationship between selected preoperative abdominal body composition and physical fitness variables, and (B) the relationship of these variables with postoperative in‐hospital major morbidity.

## METHODS

This study was designed using the STROBE Cohort Statement.

**FIGURE 2:**
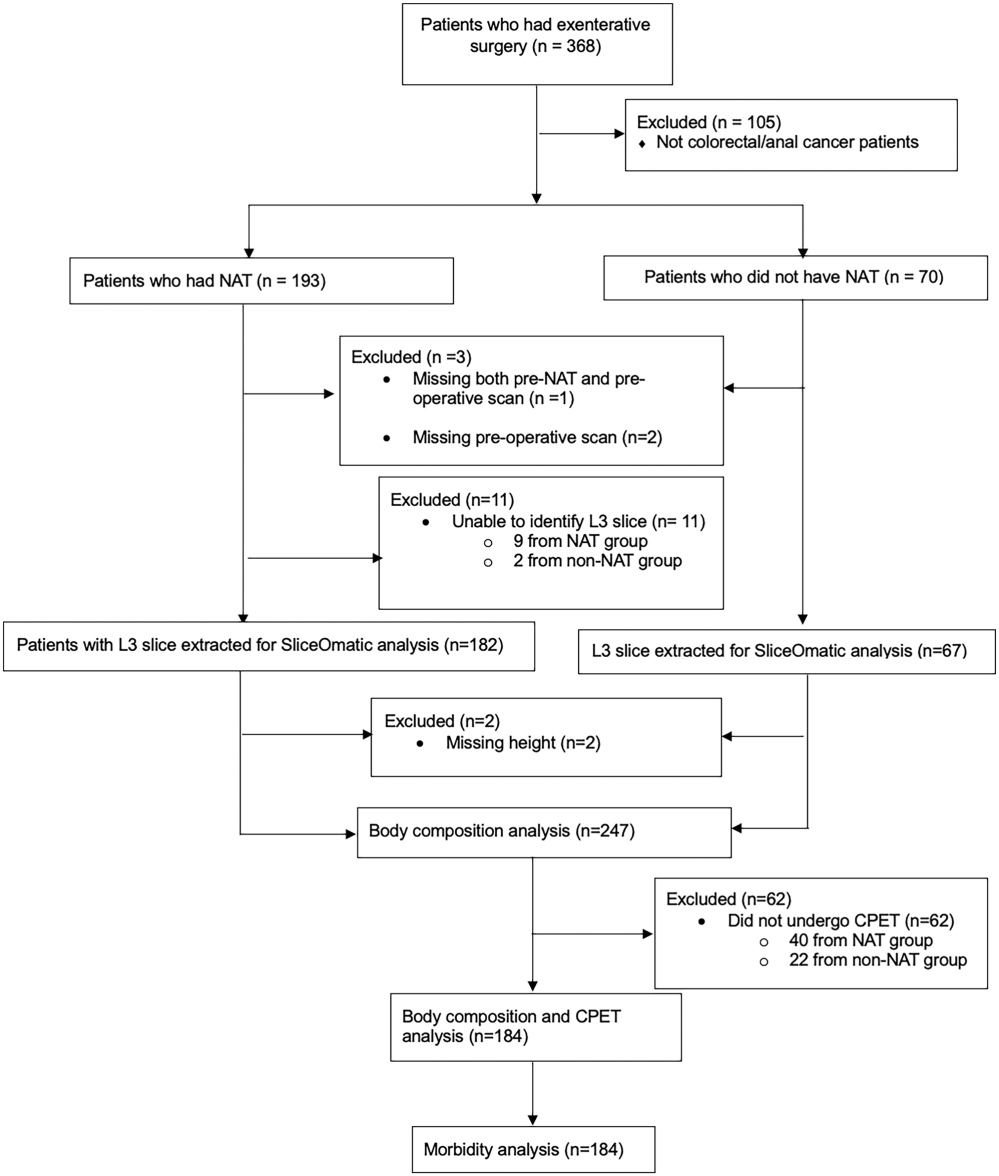
Inclusion/exclusion flow diagram.

The Southampton Complex Cancer Bioresource (REC number 18/EE/0027, clinical trials registration – NCT05219058) is a prospectively maintained data repository of patients undergoing exenterative surgery in a large quaternary complex cancer centre on the south coast of England with a catchment area of >8 million. A consecutive cohort of locally advanced and recurrent cancer patients who underwent exenterative surgery with curative intent between January 2011 and October 2023 was identified (Figure 2). Exenterative surgery was defined as *en bloc* removal of advanced or recurrent cancers either in the pelvis or abdominal cavity by partial or total resection of contiguously affected organs and/or structures [1]. The type of exenteration was defined using the externally validated UK Pelvic Exenteration Network (UKPEN) lexicon [20] and recent work published by the same group [21]. Neoadjuvant treatment was defined as any chemotherapy, radiotherapy, or chemoradiotherapy prior to this specific exenteration.

### Body composition

Using Sectra Picture Archiving and Communication System, the relevant portal venous phase CT abdomen pelvis scans were exported and then stored anonymously. Scans were imported into RadiAnt DICOM viewer to systematically extract the L3 slice following a predefined protocol. The selected L3 slice was analysed in SliceOmatic (TomoVision, Magog, Canada version 5.0) using the Alberta protocol [22]. Total cross‐sectional areas (cm^2^) of each tissue were height adjusted to produce L3 index values (SMI, VATI, SATI, IMATI) (cm^2^/m^2^). The radiation attenuation of each tissue was also assessed (SM‐RA, VAT‐RA, SAT‐RA, IMAT‐RA) (HU). Cropped scans (defined as scans with missing total surface area and SAT measurements) were included if SMI and SM‐RA measures were intact. Reproducibility of L3 slice selection and body composition analysis was validated using inter‐ and intra‐observability agreements using Bland–Altman plots. Low SMI and low SM‐RA were defined using Martin et al.'s threshold [23] (Table 1). All data except the comparison of body composition before and after NAT was taken from the CT scan immediately prior to surgery.

**TABLE 1:** Low SMI and low SM‐RA defined by Martin et al.'s threshold.

BMI category (kg/m^2^)	SMI (cm^2^/m^2^)		SM‐RA (HU)	
	Male	Female	Male	Female
<25.0	<43	<41	<41	<41
≥25	<53	<41	<33	<33

Abbreviations: BMI, Body Mass Index; SMI, skeletal muscle index; SM‐RA, skeletal muscle radiation attenuation.

### CPET

Patients underwent standardised CPET before surgery in accordance with perioperative guidance [24]. Assessors were blinded to body composition measurements and clinical outcomes. VO_2_ peak and AT were reported as absolute values (litres min^−1^) and indexed to bodyweight (mL kg^−1^ min^−1^). PPO (W kg^−1^) was defined as the maximum work rate achieved during a continuous ramped CPET protocol indexed to patient bodyweight [16]. Patients who had not undergone preoperative CPET were excluded. High‐risk groups were defined as VO_2_ AT <10.1 mL kg^−1^ min^−1^, VO_2_ peak <16.7 mL kg^−1^ min^−1^, PPO <1.5 W kg^−1^, and V_*E*_/VCO_2_ slope > 34, as failure to meet these thresholds confers additional risk in cancer patients undergoing major surgery [16].

### Morbidity

In‐hospital complications were categorised using the Clavien‐Dindo classification, with 3a or above indicating major morbidity. Morbidity data were collected prospectively by dedicated research staff blinded to body composition and CPET data.

### Statistical analysis

At 80% power and at 95% significance, a sample size of 427 patients would be required to detect significance, based on data taken from unpublished work by LM. This work had similar methodology and aims and showed major postoperative in‐hospital morbidity rates of 9.9% in the low SM‐RA group and 4.9% in the high SM‐RA group. This calculation was used to plan this pilot cohort study.

Data were analysed using IBM SPSS statistics and presented at a 5% significance level with a 95% confidence interval (95% CI). Comparative analysis of related groups used a paired t‐test or Wilcoxon signed‐rank test. Comparative analysis of two independent groups used an independent *t*‐test or Mann–Whitney U test. The analysis of the association of categorical variables used the Chi‐square test or Fisher's exact test.

Associations between body composition variables and VO_2_ AT, VO_2_ peak, and PPO were tested using linear regression. Associations between high‐risk categories of SMI, SM‐RA, and VO_2_ AT, VO_2_ peak, V_*E*_/VCO_2_, and PPO and major morbidity were tested using logistic regression models constructed using directed acyclic graphs (DAG) (Figures S4.0–S4.6). Variables significant in univariate analysis were ordered by increasing *p*‐values and added stepwise to a multivariate model. Variables were either entered or removed at the 0.05 and 0.1 probability levels respectively. Model fit was tested using the F‐test or Nagelkerke *r* ^2^. A receiver operating characteristic (ROC) curve was plotted and the area under the curve (AUC) was calculated for the multivariate major morbidity model.

## RESULTS

Whole cohort patient demographic data are presented in Table 2. Median time between preoperative scan and surgery was 38 days. No significant difference was found in L3 slice selection between inter‐ and intra‐observers. Significant intra‐observer differences were found for SAT and SM‐RA (*p* = 0.015 and *p* = 0.033 respectively), despite ICC > 0.983 for all results. Significant inter‐observer differences were found for VAT alone (*p* = 0.008; ICC > 0.997). Bland–Altman plots and agreement tables are displayed in Figures S1.0–S1.3 and Tables S1.0–S1.1.

**TABLE 2:** Whole cohort patient characteristics and morbidity data.

			Complete cohort (*n* = 247)	
			Number of patients (%)	Mean (±SD)/median (IQR)
Age				60.4 (52.2–69.2)
Sex (Male)			142 (57.5)	
Weight (kg)				78.3 (68.0–90.2)
Height (m)				1.7 ± 0.09
BMI (kg/m^2^)				26.4 (23.6–30.5)
Cancer location	Colorectal		238 (96.4)	
	Anal		9 (3.6)	
Cancer type	Locally advanced cancer		153 (62.4)	
	Locally recurrent cancer		87 (35.5)	
	Re‐recurrent rectal cancer		4 (1.6)	
	Other		1 (0.4)	
TNM staging	T stage	T2	5 (2.1)	
		T3	59 (24.4)	
		T4	91 (37.6)	
		Recurrence	87 (36.0)	
	N stage	N0	36 (14.8)	
		N1	54 (22.1)	
		N2	67 (27.5)	
		Recurrence	87 (35.7)	
Metastatic disease			64 (26.0)	
NAT			177 (72.0)	
	Chemotherapy alone		37 (15.0)	
	Radiotherapy alone		9 (3.7)	
	Chemoradiotherapy		131 (53.3)	
Historic systemic chemotherapy			45 (18.4)	
Historic pelvic radiotherapy			66 (26.9)	
Previous abdominal surgery			176 (71.3)	
ASA Grade	1		9 (3.8)	
	2		128 (53.6)	
	3		100 (41.8)	
	4		2 (0.8)	
Preoperative Hb (g/L)				131.6 (±15.0)
Exenteration Type	High‐complexity supralevator		107 (43.3)	
	High‐complexity infralevator		68 (27.5)	
	Conventional supralevator		31 (12.6)	
	Conventional infralevator		21 (8.5)	
Resection margin status	R0		209 (84.7)	
	R1		38 (15.4)	
Index admission Clavien‐Dindo Score	No complications	0	93 (38.4)	
	Major	3a	31 (12.8)	
		3b	9 (3.7)	
		4a	3 (1.2)	
		4b	6 (2.5)	
		5	2 (0.8)	

*Note*: Missing weight, BMI, metastatic, NAT, emergency admission data (*n* = 1), missing smoking status, cancer type, historic pelvic therapy (*n* = 2), missing N stage (*n* = 3), missing T stage (*n* = 5), missing ASA grade (*n* = 8).

Abbreviations: ASA, American Society of anaesthesiologists; Hb, haemoglobin; TNM, tumour node metastasis classification.

### Body composition

Whole cohort body composition data can be found in Table S2.0.

Preoperatively, 61.5% (32.4% males, 29.1% females) and 37.2% (19.4% males, 17.8% females) of the cohort were classified as low SMI and low SM‐RA respectively.

72% of patients underwent NAT, following which median SMI decreased in females (−0.7 cm^2^/m^2^; *p* = 0.011) and males (−1.0 cm^2^/m^2^; *p* = 0.216). Median SM‐RA decreased in males (−2.2 HU; *p* = 0.025) but increased in females by (+0.7 HU; *p* = 0.797).

Low SMI and low SM‐RA statuses were not significantly associated before or after NAT (*X* ^2^ = 0.309, *p* = 0.579, and *X* ^2^ = 0.838, *p* = 0.360 respectively).

### CPET

About 184 patients underwent CPET prior to surgery. Median VO_2_ peak and VO_2_ AT were 19.8 (IQR = 3.2) and 11.2 (IQR = 7.1) mL kg^−1^ min^−1^ respectively. Detailed whole cohort CPET data can be found in Table S3.0. All measures of physical fitness were significantly worse in low SMI or low SM‐RA patients (Table 3).

**TABLE 3:** CPET data by low SMI or low SM‐RA status.

	Low SMI			Low SM‐RA		
	Mean (±SD)/median (IQR)		*p*‐value	Mean (±SD)/median (IQR)		*p*‐value
	Yes (*n* = 105)	No (*n* = 79)		Yes (*n* = 62)	No (*n* = 122)	
VO_2_ AT (Litres min^−1^)	0.9 (±0.2)	1.0 (±0.2)	**0.001** *	0.9 (±0.3)	0.9 (±0.2)	0.110
VO_2_ AT (ml kg^−1^ min^−1^)	10.9 (3.3)	11.4 (3.5)	0.126	10.4 (3.5)	11.6 (3.4)	**0.001** *
Work rate AT	56.0 (29.0)	66.0 (34.0)	**0.016** *	55.0 (27.0)	62.0 (29.0)	**0.022** *
VO_2_ peak (Litres min^−1^)	1.5 (±0.5)	1.8 (±0.4)	**0.001** *	1.5 (±0.5)	1.7 (±0.45)	**0.033** *
VO_2_ peak (ml kg^−1^ min^−1^)	18.1 (7.3)	20.8 (7.6)	**0.010** *	17.7 (5.4)	20.9 (7.9)	**0.001** *
Work rate peak	122.0 (63.0)	158.0 (50.0)	**0.001** *	122.0 (62.0)	144.0 (59.0)	**0.020** *
PPO (W kg^−1^)	1.6 (0.7)	1.8 (0.7)	0.106	1.5 (0.6)	1.7 (0.7)	**0.008** *

Abbreviations: AT, anaerobic threshold; Low SMI, low skeletal muscle index; Low SM‐RA, low skeletal muscle radiation attenuation; peak, peak exercise; PPO, peak power output; VO_2_, oxygen uptake. *n* = 184.

* Significant at 5% level.

In multivariate analysis, SMI was predictive of VO_2_ AT (B = 0.013, 95% CI (0.009–0.010), *p* = 0.001) and VO_2_ peak (B = 0.015 95% CI (0.006–0.024), *p* = 0.002), and SM‐RA was predictive of PPO (B = 0.014 95% CI (0.002–0.025), *p* = 0.017). Linear regression models can be found in Tables S4.0–S4.1.

### Morbidity

A total of 21% of patients overall experienced postoperative major morbidity. Low VO_2_ peak, AT, and PPO groups, were significantly associated with increased major morbidity in univariate analysis (Table 4). Neither low SMI nor SM‐RA were associated with postoperative morbidity. In multivariate analysis only reduced PPO was associated with an increased risk of major morbidity (OR = 2.6 95% CI(0.2–5.5), *p* = 0.012), with an AUC of 0.672. The logistic regression model and accompanying ROC curve can be found in Table S5.0 and Figure S3.0.

**TABLE 4:** Proportion of major morbidity events in selected high‐risk body composition and CPET groups.

*n* (%)	*n* (%)	OR (95% CI)	*p*‐value
Low SMI	High SMI		
28 (26.7)	14 (17.7)	1.7 (0.8–3.5)	0.152
Low SM‐RA	High SM‐RA		
17 (27.4)	25 (20.5)	1.5 (0.7–3.0)	0.290
Low VO_2_ peak	High VO_2_ peak		
20 (35.8)	22 (17.2)	2.7 (1.3–5.5)	**0.006** *
Low VO_2_ AT	High VO_2_ AT		
19 (31.7)	23 (18.5)	2.1 (1.0–4.1)	**0.047** *
Low PPO	High PPO		
22 (34.9)	20 (16.5)	2.7 (1.3–5.5)	**0.005** *
High V_*E*_/VCO_2_ Slope	Low V_*E*_/VCO_2_ Slope		
6 (23.1)	36 (22.8)	1.0 (0.3–2.7)	0.974

*Note*: Low VO_2_ AT <10.1 mL kg^−1^ min^−1^, Low VO_2_ peak <16.7 mL kg^−1^ min^−1^, Low PPO < 1.5 W kg^−1^, and High V_*E*_/VCO_2_ > 34. *n* = 184.

Abbreviations: AT, anaerobic threshold; peak, peak exercise; PPO, peak power output; V_E_/VCO_2_, ventilation/carbon dioxide production; VO_2_, oxygen uptake; VO_2_/work rate slope, oxygen uptake/work rate relationship.

* Significant at 5% level.

## DISCUSSION

To the best of our knowledge this is the first report of preoperative abdominal body composition and physical fitness in patients undergoing exenterative surgery for locally advanced and locally recurrent cancers. We found that 61% and 37% of our patients had low SMI and low SM‐RA respectively compared with a pooled incidence of 34% of low SMI in unselected colorectal cancer populations [8].

Body composition variables were associated with physical fitness in multivariate analysis with SM‐RA predictive of PPO, SMI predictive of absolute VO_2_ AT, and alongside age, male sex, and SAT‐RA, were predictive of absolute VO_2_ peak. Physical fitness variables were moderately predictive of major morbidity with low PPO (<1.5 W.kg^−1^) predicting an increased risk of major morbidity in multivariate analysis. Despite body composition being associated with physical fitness, body composition variables were not predictive of major postoperative morbidity.

Our patient cohort was fitter than populations of unselected colorectal cancer patients with a higher relative VO_2_ at peak [19, 25], and VO_2_ at, and peak work rate [19], and this may explain why physical fitness, not body composition was more predictive of major morbidity in this cohort. Our fitter cohort is likely reflective of not all patients undergoing NAT, which adversely impacts physical fitness [6]. It may also reflect selection bias, as patients with poor physical fitness in isolation or after neoadjuvant treatment did not proceed to exenterative surgery.

In agreement with published literature [26], male SMI and SM‐RA, and female SMI decreased following NAT. As expected, low SMI or low SM‐RA patients had significantly worse physical fitness (Table 3). This suggests that muscle quality in addition to muscle mass is an important determinant of physical fitness as has been reported previously [27]. SM‐RA is a measure of myosteatosis, characterised by reduced muscle quality due to fat infiltration. It is likely that the increased muscle lipid content and related mitochondrial dysfunction result in decreased oxygen consumption and extraction by the muscle during exercise [27]. Muscle mass (SMI) is associated with AT and VO_2_ peak because mitochondrial oxygen uptake is proportional to the mass of exercising muscle.

Male sex and age both impact exercise capacity because of their impact on muscle mass and consequently oxygen uptake. Males have on average higher muscle mass than females and muscle mass decreases in both sexes with age as does maximum cardiac output [28]. Our multivariate model was in agreement with work published by Berkel et al., who reported that SMI is significantly associated with absolute VO_2_, and SM‐RA with relative values in colorectal cancer patients [19], and a hepato‐pancreato‐biliary cohort which reported associations between relative VO_2_ and SM‐RA [27]. There was no association between low SMI and low SM‐RA in our cohort, suggesting that muscle quality is independent of muscle quantity and that reduced muscle quality may impact function without a reduction in muscle mass. This finding is also in agreement with a prospective cohort study which showed that a decline in strength was much more rapid than the concomitant loss of muscle mass [29].

A significantly lower proportion of patients (17%) experienced in‐hospital postoperative major morbidity, when compared with a recent cohort of pelvic exenteration patients [30]. We found no association between body composition and major morbidity which concurs with the only previously published cohort of pelvic exenteration patients [12]. This is in contrast to cohorts of unselected colorectal cancer [31] and major abdominal surgical patients [32], where body composition was associated with both morbidity and mortality. Physical fitness variables, in contrast, were associated with major morbidity, with a significant increase in major morbidity in three out of four high‐risk CPET parameters (Table 4). Chemoradiotherapy, low VO_2_ peak, and low PPO were associated with major morbidity in univariate analysis. Only low PPO was significantly associated with major morbidity in multivariate analysis, identifying it as a new potential prognostic factor in this cohort. However, with an AUC of 0.672, this multivariate model only shows moderate discriminative ability. It was recently reported to be associated with postoperative complications after major cancer surgery [16]. Technically, it is easier to determine than either VO_2_ peak or the anaerobic threshold as it can be measured without measuring gas exchange during a maximal exercise test and so requires less interpretation expertise (although ECG and blood pressure monitoring are still required for safety). Our cohort confirms the potential clinical utility of this variable to identify a group of patients at high risk of postoperative morbidity [16]. A high V_*E*_/VCO_2_ slope was not associated with major morbidity, despite its association with morbidity in an unselected colorectal cancer population [33]. Our study confirms the utility of CPET over CT body composition analysis for the prediction of postoperative morbidity in the pelvic exenteration population.

## STRENGTH AND LIMITATIONS

To the best of our knowledge this is the first report of CPET and CT body composition in a cohort of pelvic exenteration patients. It is however a single‐centre cohort, and confirmation in an adequately powered, multicentre setting to ascertain external validity would be valuable. We acknowledge a selection bias and confounding by indication, as only patients who had technically curable cancers on imaging review and who were subjectively assessed as fit for exenteration by the surgical team underwent CPET and were included in the cohort. Furthermore, patients attend a shared‐decision‐making high‐risk clinic if found objectively unfit and since October 2023 perioperative and surgical management is altered with the introduction of formal exercise prehabilitation. Furthermore, the small cohort of patients who underwent CPET was because, before 2018, the perioperative medicine service at Southampton was not a funded NHS service. As a result, not all major surgical patients were tested, particularly those referred from outside Hampshire.

We did not report IMAT which is a more robust measure of myosteatosis, that unlike SM‐RA is not impacted by the accumulation of oedema [34]. Furthermore, CT measures of body composition are impacted by the contrast media and slice thickness used, and as CT protocols change over time, this may have impacted body composition analysis. Although the significance values produced in the inter‐observer agreement for SM‐RA were significant, the differences were within the confidence interval, suggesting that although there is a statistically significant difference, the magnitude of this difference may not be practically significant and may not have an effect on results [35]. The DAGs created highlight the absence of key variables that affect physical fitness and body composition such as ethnicity and socioeconomic status, which therefore remained unadjusted in the models.

Based on the findings of this study, physical fitness measured by CPET may be predictive of major morbidity in this cohort. The association of fitness and body composition necessitates further evaluation and external validation in a larger patient cohort, specifically interrogating their combined role in morbidity prediction and as a target for prehabilitation interventions. Furthermore, the predictive ability of other functional testing such as the 6‐minute walk test, sit‐to‐stand testing and other functional questionnaires to supplement CPET and/or body composition is worth exploring. Research can now be directed towards defining an optimal physical fitness prehabilitation programme, comprising aerobic capacity or resistance training, to improve patient outcomes and improve morbidity risk in pelvic exenteration patients. However, the evidence base for prehabilitation is still growing. A recent exercise programme, using high‐intensity aerobic training, was shown to improve physical fitness, specifically VO_2_ at AT, in a locally advanced rectal cancer cohort [36] and a recent community‐based prehabilitation programme consisting of both high‐intensity aerobic training and resistance training was shown to reduce the risk of postoperative morbidity in high‐risk colorectal cancer patients [37]. Future work directed towards evaluating the efficacy of prehabilitation interventions in a pelvic exenteration cohort would be beneficial.

## CONCLUSION

Our study supports the evidence that CPET may be useful for identifying patients at high risk of postoperative morbidity after pelvic exenteration surgery. Despite being associated with fitness, CT body composition variables were not associated with postoperative morbidity in this cohort. Further research is warranted to clarify the relationships between other functional tests, CPET, and body composition to predict morbidity in this patient group.

## AUTHOR CONTRIBUTIONS

**Marina Looby:** Methodology; investigation; writing – original draft; software; formal analysis. **Lewis Matthews:** Methodology; validation; writing – review and editing; software. **Charles T. West:** Data curation; writing – review and editing. **Kashuf Khan:** Writing – review and editing. **Gillian Ansell:** Writing – review and editing. **Kathryn Donovan:** Writing – review and editing. **Laura Wood:** Writing – review and editing. **Patrick Tapley:** Writing – review and editing. **Rhys Lewis:** Writing – review and editing. **Kate Stoddard:** Writing – review and editing. **Michael P. W. Grocott:** Writing – review and editing. **Sandy Jack:** Writing – review and editing. **Hideaki Yano:** Writing – review and editing. **Denny Levett:** Writing – review and editing; supervision. **Alex Mirnezami:** Writing – review and editing; supervision. **Malcolm A. West:** Writing – review and editing; supervision; funding acquisition; conceptualization; methodology; resources.

## CONFLICT OF INTEREST STATEMENT

The authors have no conflict of interest to declare.

## ETHICS STATEMENT

Southampton Complex Cancer Bioresource (REC number 18/EE/0027).

## PATIENT CONSENT STATEMENT

All patients consented to be in the database that was covered by the ethics approval statement.

## Supporting information


Data S1:

## Data Availability

The data that support the findings of this study are available from the corresponding author upon reasonable request.
