# Supplementary material for: Physical fitness and body composition assessments in advanced cancer patients undergoing exenterative surgery – A pilot cohort study
Source: Colorectal Dis. 2025 Nov 18;27(11):e70298. doi: 10.1111/codi.70298 (PMC12626906; doi:10.1111/codi.70298)

**Supplementary figures and tables**

**Intra- and Inter-observability agreement for L3 slice selection**

*Intra-observability agreement:*

*Supplementary Figure 1.0****:*** Intra-observer Bland-Altman:
The line represents the mean difference. No statistically significant differences between intra-observer parameters. With mean difference being 0 consistently, 95% agreement limits, SEM and SD could not be computed. P-value = 1, determined from paired samples t-test.

**
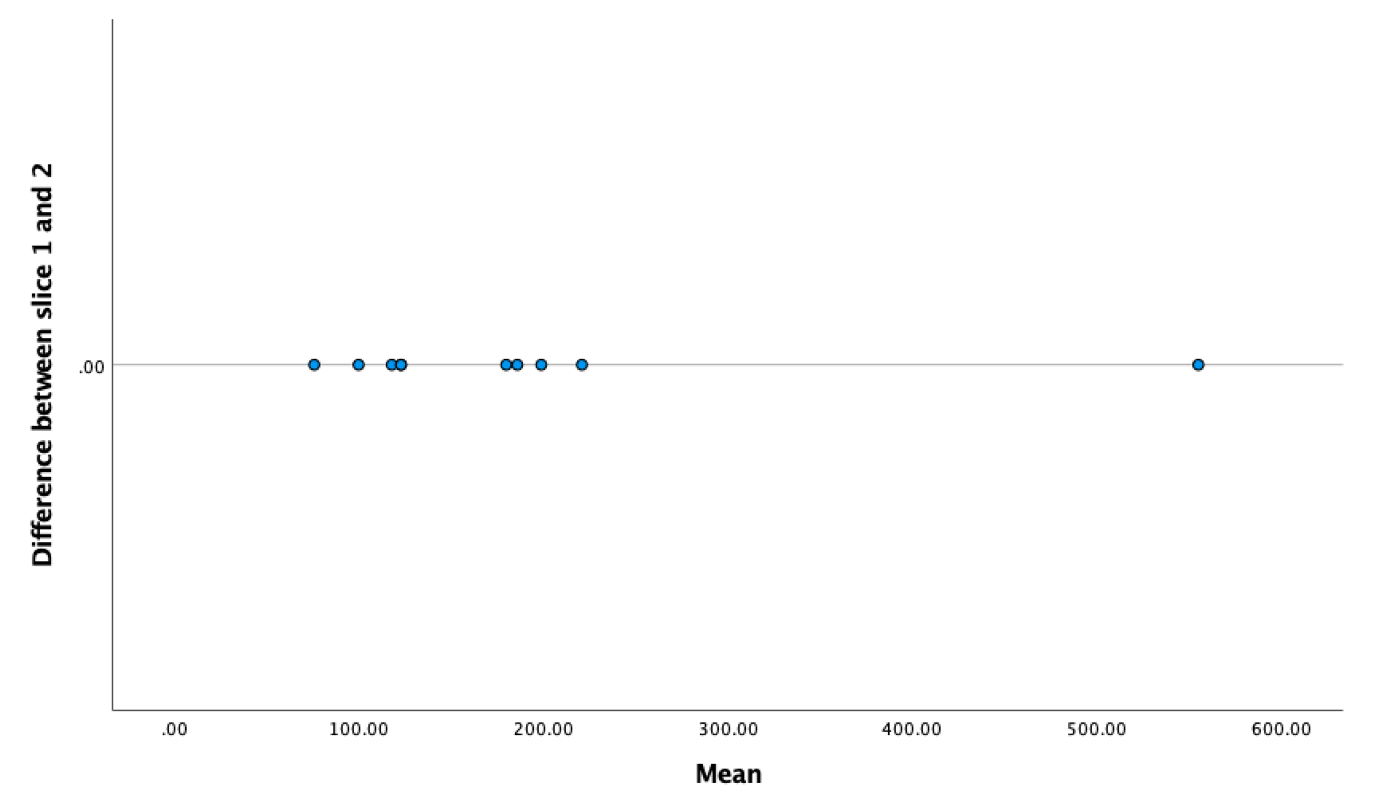
**

*Inter-observability agreement:*

*Supplementary Figure 1.1****:*** Inter-observer Bland-Altman:
The line represents the mean difference. No statistically significant differences between intra-observer parameters. With mean difference being 0 consistently, 95% agreement limits, SEM and SD could not be computed. P-value = 1, determined from paired samples t-test.

**
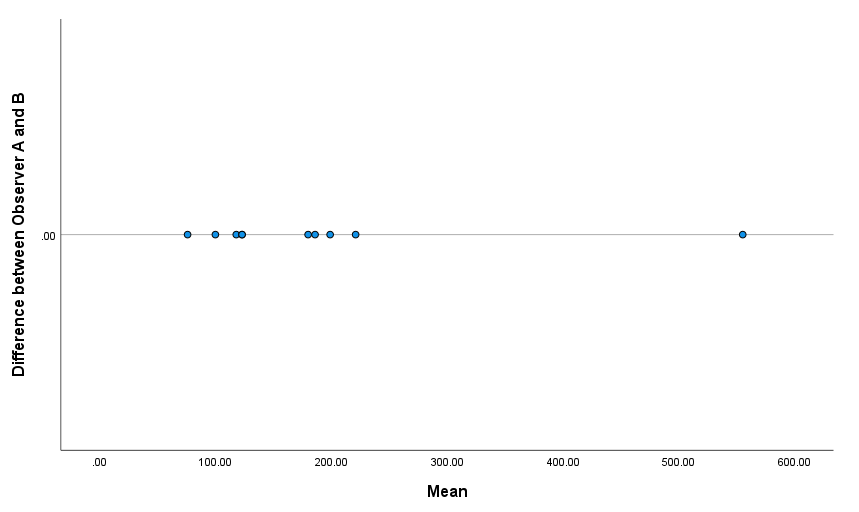
**

**Intra- and Inter-observability agreement for L3 slice analysis**

*Intra-observability agreement:*

Supplementary Table 1.0: Values for intra-observability agreement.

|  | Mean (±SD) / Median (IQR) difference | P value | ICC (95%Cl) |
| --- | --- | --- | --- |
| SAT (cm^2^) | 1.30 (2.10) | **0.015*** _a_ | 0.999 (0.995; 1.00) |
| VAT (cm^2^) | -0.99 (±3.21) | 0.381 _b_ | 1.00 (0.999; 1.00) |
| IMAT (cm^2^) | -0.97 (±1.84) | 0.152 _b_ | 0.983 (0.923; 0.996) |
| SM (cm^2^) | -0.88 (±1.39) | 0.095 _b_ | 0.999 (0.996; 1.000) |
| SM-RA (HU) | -0.90 (1.60) | **0.033*** _a_ | 1.00 (0.996; 1.000) |
| a = Wilcoxon signed ranks test, b = paired samples t-test. * = significant at the 5% level.  SAT = Subcutaneous Adipose Tissue, VAT = Visceral Adipose Tissue, IMAT = Intramuscular Adipose Tissue, SM = Skeletal Muscle, SM-RA = Skeletal Muscle Radiation Attenuation. | | | |

*Supplementary Figure 1.2****:*** Intra-observer Bland-Altman 95% agreement limits:
The lines represent the mean difference and the 95% agreement limits (± 1.96 SD). All units are cm^2^, except mean muscle attenuation which is hounsfield units. No statistically significant differences between intra-observer parameters; VAT, IMAT and, SM.


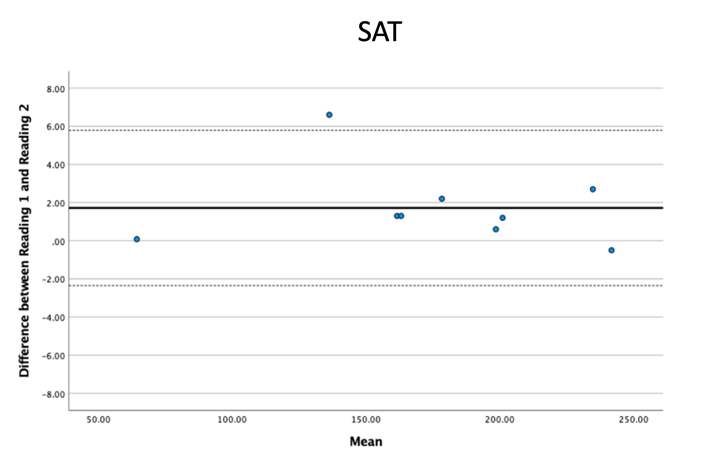


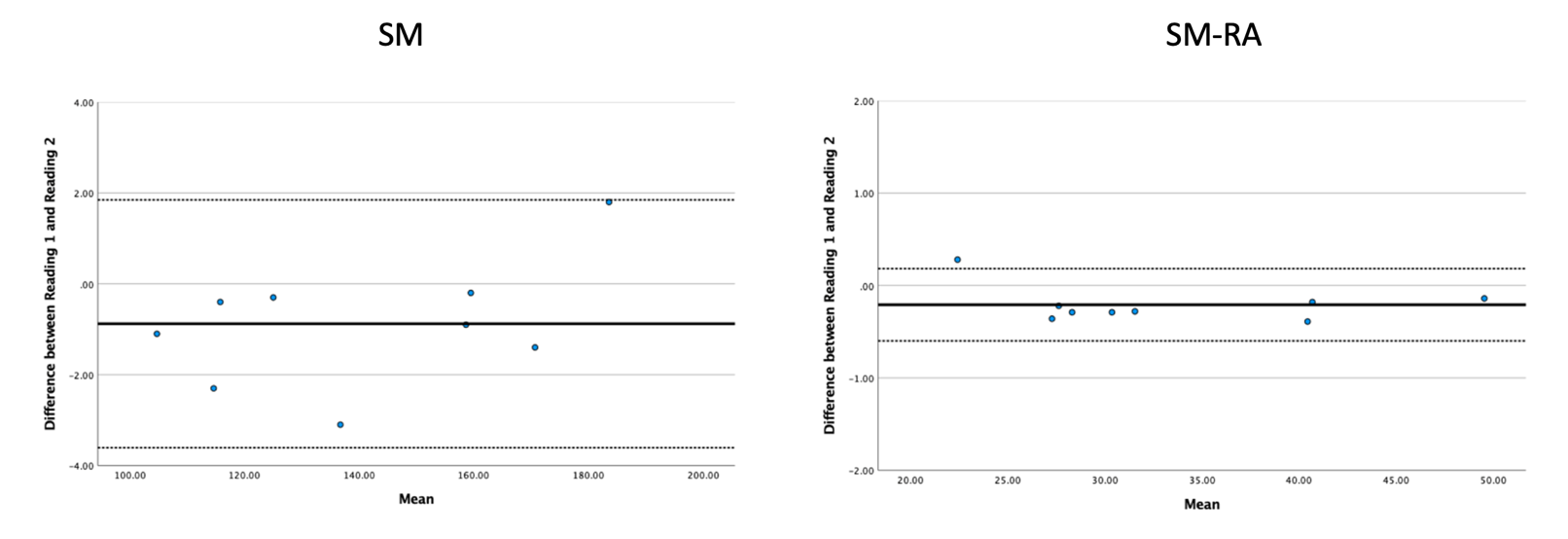


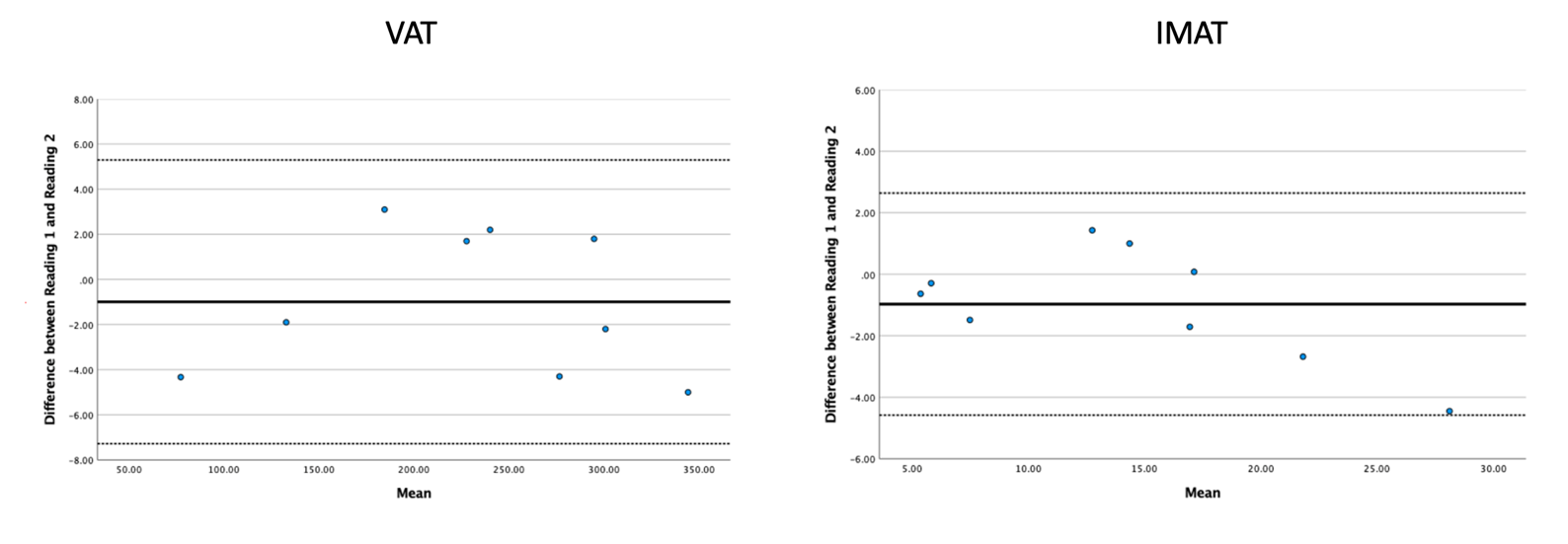


**Inter-observability agreement with previous observer:**

Supplementary Table 1.1: Values for inter-observability agreement with previous observer.

|  | Mean (±SD) difference | P value | ICC (95%Cl) |
| --- | --- | --- | --- |
| SAT (cm^2^) | 1.19 (±1.92) | 0.684_b_ | 0.999 (0.987-1) |
| VAT (cm^2^) | 12.35 (±5.57) | **0.008*_b_** | 0.997 (0.973-1) |
| IMAT (cm^2^) | 0.25 (±1.75) | 0.757_b_ | 0.908 (0.366-0.990) |
| SM (cm^2^) | 1.22 (±2.47) | 0.331_b_ | 0.990 (0.911-0.999) |
| SM-RA (HU) | -0.22 (±0.59) | 0.46_b_ | 0.995 (0.950-0.999) |
| * = significant at 5% level.  SAT = Subcutaneous Adipose Tissue, VAT = Visceral Adipose Tissue, IMAT = Intramuscular Adipose Tissue, SM = Skeletal Muscle, SM-RA = Skeletal Muscle Radiation Attenuation, HU = Hounsfield Unit. | | | |

*Supplementary Figure 1.3****:*** Inter-observer Bland-Altman 95% agreement limits for previous observer: The lines represent the mean difference and the 95% agreement limits (± 1.96 SD). Observer B is the previous observer. All units are in cm^2^, except mean muscle attenuation which is in hounsfield units. Statistically significant differences between intra-observer VAT.

**
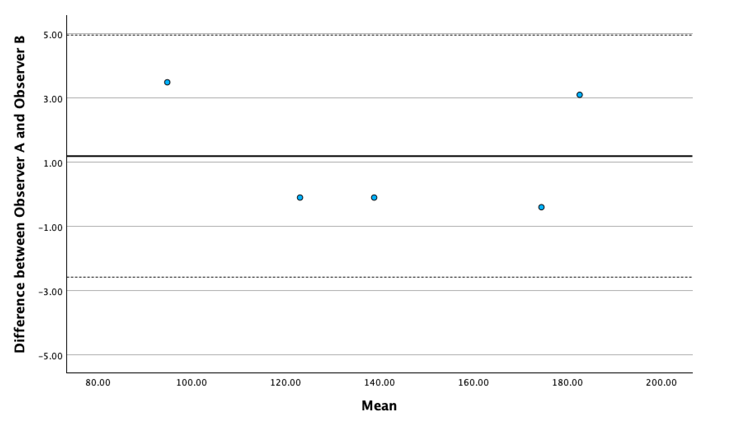
**

**
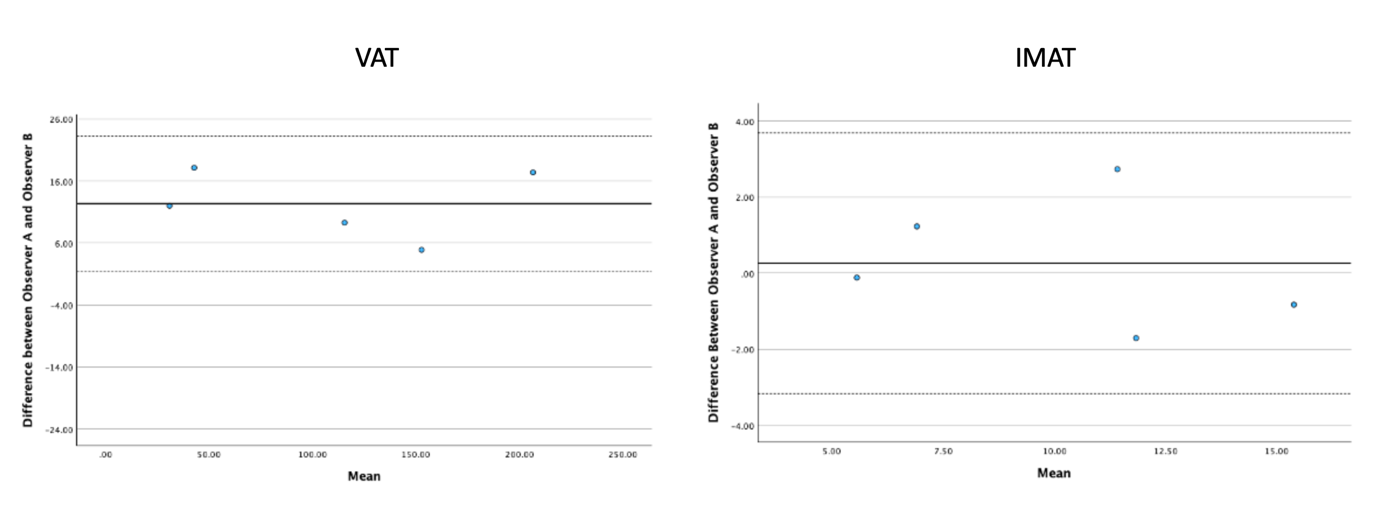
**

**
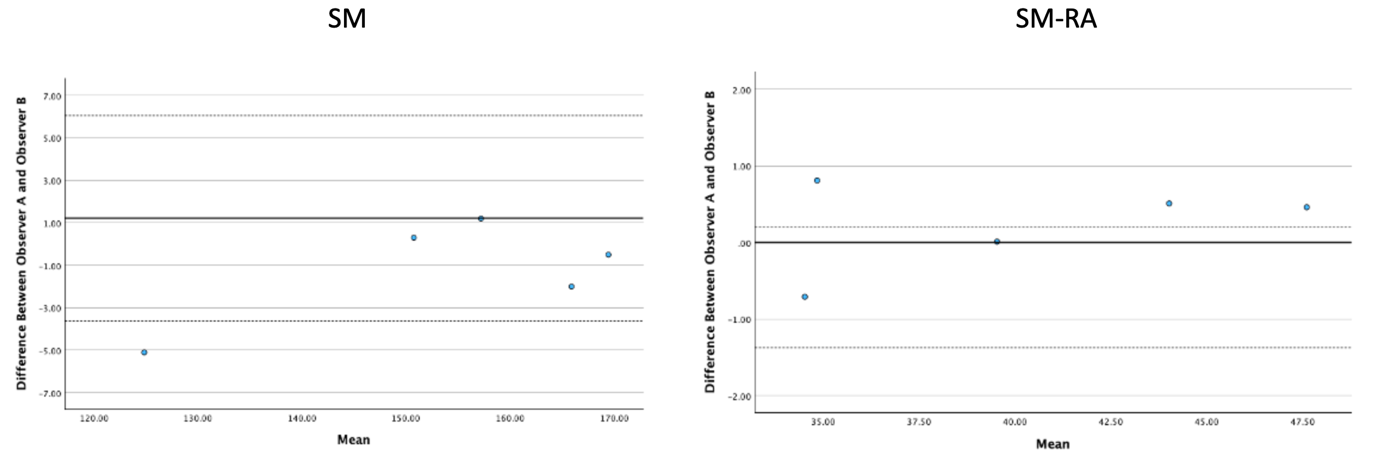
**

**Supplementary** Table 2.0: Sex specific whole cohort body composition data.

|  | Mean (± SD) / Median (IQR) | | | p-value |
| --- | --- | --- | --- | --- |
|  | Overall (n=247) | Male (n=142) | Female (n=105) |  |
| SATI (cm^2^/m^2^) | 59.0 (42.7-89.8) | 50.9 (36.4-65.4) | 85.4 (51.8-119.0) | **0.001*** |
| VATI (cm^2^/m^2^) | 46.1 (24.8-69.5) | 57.0 (34.7-79.3) | 35.8 (15.4-56.2) | **0.001*** |
| IMATI (cm^2^/m^2^) | 3.8 (2.3-5.7) | 3.7 (1.9-5.5) | 3.1 (1.3-4.7) | 0.490 |
| SMI (cm^2^/m^2^) | 43.7 (38.0-50.4) | 47.2 (41.9-52.5) | 38.0 (33.9-42.1) | **0.001*** |
| SAT-RA (HU) | -101.8 (-106.8 - -96.0) | -100.6 (-105.8 - -95.4) | -102.9 (-108.3--97.5) | 0.062 |
| VAT-RA (HU) | -93.0 (-97.8 - -86.3) | -93.5 (-98.53 - -88.5) | -92.0 (-99.1- -84.9) | 0.114 |
| IMAT-RA (HU) | -62.5 (-67.6 - -58.7) | -62.8 (-67.2 - -58.4) | -62.4 (-67.1- -57.7) | 0.717 |
| SM-RA (HU) | 38.3 (±8.4) | 38.6 (±7.8) | 38.02 (±9.1) | 0.617 |
| SATI and SAT-RA (n=225). * = significant at 5% level.  SATI = Subcutaneous Adipose Tissue Index, VATI = Visceral Adipose Tissue Index, IMATI = Intramuscular Adipose Tissue Index, SMI = Skeletal Muscle Index, SAT-RA = Subcutaneous Adipose Tissue Index, VAT-RA = Visceral Adipose Tissue Index, IMAT-RA = Intramuscular Adipose Tissue Index, SM-RA = Skeletal Muscle Radiation Attenuation, HU = Hounsfield Unit. | | | | |

Supplementary Table 3.0: Cardiopulmonary exercise testing data. (n=184)

|  | Mean (±SD) / Median (IQR) |
| --- | --- |
| VO_2_ AT (Litres.min^-1^) | 0.9 (±0.2) |
| VO_2_ AT (ml.kg^-1^.min^-1^) | 11.2 (3.2) |
| V*_E_*/VCO_2_ AT | 29.7 (5.0) |
| Work rate AT | 60.0 (32.0) |
| VO_2_ peak (Litres.min^-1^) | 1.6 (±0.5) |
| VO_2_ peak (ml.kg^-1^.min^-1^) | 19.8 (7.1) |
| V*_E_*/VCO_2_ peak | 34.3 (5.4) |
| Work rate peak | 135.0 (58.0) |
| VO_2_/work rate slope (ml.min^-1^W^-1^) | 9.6 (1.2) |
| V*_E_*/VCO_2_ slope | 28.7 (6.6) |
| PPO (W.kg^-1^) | 1.7 (0.7) |
| VO_2_ = Oxygen Uptake, AT = Anaerobic Threshold, V*_E_*/VCO_2_ = Ventilation / Carbon Dioxide Production, peak = Peak Exercise, VO_2_/work rate slope = Oxygen Uptake / Work Rate Relationship, PPO = Peak Power Output. | |

Supplementary Table 4.0: Body composition and VO_2_ AT and VO_2_ peak linear regression models

| VO_2_ AT (Litres.min^-1^) | | | | | VO_2_ Peak (Litres.min^-1^) | | | |
| --- | --- | --- | --- | --- | --- | --- | --- | --- |
|  | Univariate | | Multivariate | | Univariate | | Multivariate | |
|  | B (95% CI) | p-value | B (95% CI) | p-value | B (95% CI) | p-value | B (95% CI) | p-value |
| Age | -0.002 (-0.005-0.001) | 0.126 |  |  | -0.009 (-0.015- -0.003) | **0.003*** | -0.012 (-0.018- -0.006) | **0.001*** |
| Sex (Male) | 0.164 (0.097-0.231) | **0.001*** | 0.180 | 0.197 | 0.304 (0.172-0.436) | **0.001*** | 0.222 (0.060-0.384) | **0.008*** |
| BMI (kg/m^2^) | 0.013 (0.006-0.019) | **0.001*** | 0.088 | 0.257 | 0.021 (0.007-0.034) | **0.002*** | -0.012 | 0.892 |
| VATI (cm^2^/m^2^) | 0.000 (0.000-0.000) | **0.161** |  |  | 0.001 (0.000-0.002) | 0.239 |  |  |
| SMI (cm^2^/m^2^) | 0.013 (0.009-0.016) | **0.001*** | 0.013 (0.009-0.017) | **0.001*** | 0.024 (0.017-0.032) | **0.001*** | 0.015 (0.006-0.024) | **0.002*** |
| IMATI (cm^2^/m^2^) | 0.005 (-0.004-0.015) | 0.282 |  |  | -0.006 (0.025-0.014) | 0.554 |  |  |
| SAT-RA (HU) | -0.004 (-0.008- -0.001) | **0.011*** | -0.129 | 0.067 | -0.10 (-0.016- -0.003) | **0.004*** | -0.008 (-0.014- -0.003) | **0.005*** |
| SM-RA (HU) | -0.001 (-0.006-0.003) | 0.567 |  |  | 0.003 (-0.005-0.012) | 0.472 |  |  |
| IMAT-RA (HU) | -0.004 (-0.008-0.00) | 0.070 |  |  | -0.005 (-0.013-0.003) | 0.249 |  |  |
| F = 39.8. | | | | | F = 16.8. | | | |
| * = significant at 5% level. | | | | | | | | |

Supplementary Table 4.1: Body composition and PPO linear regression model

| PPO (W.kg^-1^) | | | | |
| --- | --- | --- | --- | --- |
|  | Univariate | | Multivariate | |
|  | B (95% CI) | p-value | B (95% CI) | p-value |
| Age | -0.006 (-0.014-0.001) | 0.090 |  |  |
| Sex (Male) | 0.125 (-0.045-0.296) | 0.149 |  |  |
| BMI (kg/m^2^) | -0.037 (-0.053-0.022) | **0.001*** | -0.027 (-0.045-0.008) | **0.005*** |
| SATI (cm^2^/m^2^) | -0.002 (-0.004-0.000) | **0.029*** | 0.010 | 0.708 |
| VATI (cm^2^/m^2^) | -0.002 (-0.003-0.000) | **0.011*** | -0.067 | 0.846 |
| SMI (cm^2^/m^2^) | -0.002 (-0.009-0.012) | 0.759 |  |  |
| IMATI (cm^2^/m^2^) | -0.032 (-0.056-0.009) | **0.007*** | 0.019 | 0.619 |
| SAT-RA (HU) | 0.001 (-0.007-0.009) | 0.810 |  |  |
| VAT-RA (HU) | 0.002 (-0.001-0.005) | 0.124 |  |  |
| SM-RA (HU) | 0.021 (0.011-0.031) | **0.001*** | 0.014 (0.002-0.025) | **0.017*** |
| IMAT-RA (HU) | 0.033 (-0.008-0.013) | 0.609 |  |  |
| F = 11.9. * = significant at 5% level. | | | | |

Supplementary Table 5.0: Logistic regression for major morbidity using high-risk categories of body composition and physical fitness variables.

|  | | Univariate | | Multivariate | |
| --- | --- | --- | --- | --- | --- |
|  |  | OR (95% CI) | p-value | OR (95% CI) | p-value |
| Age | | 1.017 (0.986-1.049) | 0.288 |  |  |
| BMI (kg/m^2^) | | 0.965 (0.899-1.035) | 0.319 |  |  |
| Smoking | | 1.084 (0.637-1.843) | 0.766 |  |  |
| T Stage 4 | | 0.520 (0.080-3.384) | 0.494 |  |  |
| NAT | Chemotherapy | 0.571 (0.190-1.715) | 0.318 |  |  |
|  | Radiotherapy | 2.667 (0.528-13.477) | 0.235 |  |  |
|  | Chemoradiotherapy | 0.391 (0.174-0.877) | **0.023*** | 0.481 (0.208-1.112) | 0.087 |
| Recurrent Cancer | | 1.351 (0.657-2.778) | 0.413 |  |  |
| Metastatic Cancer | | 0.672 (0.295-1.528) | 0.343 |  |  |
| R1 | | 1.496 (0.170-13.174) | 0.716 |  |  |
| Emergency Admission | | 1.763 (0.504-6.173) | 0.375 |  |  |
| Blood Loss (ml) | | 1.000 (1.000-1.001) | 0.001 |  |  |
| Pre-op Hb (g/L) | | 0.978 (0.954-1.002) | 0.078 |  |  |
| Sarcopenic (cm^2^/m^2^) | | 1.688 (0.821-3.474) | 0.155 |  |  |
| Myosteatotic (HU) | | 1.466 (0.720-2.982) | 0.291 |  |  |
| Low VO_2_ Peak (ml.kg^-1^min^-1^) | | 2.677 (1.311-5.466) | **0.007*** |  |  |
| Low VO_2_ AT (ml.kg^-1^min^-1^) | | 2.035 (1.003-4.130) | **0.049*** |  |  |
| High V*_E_*/VCO_2_ slope | | 1.017 (0.031-2.003) | 0.974 |  |  |
| Low PPO (W.kg^-1^) | | 2.710 (1.338-5.490) | **0.006*** | 2.597 (1.236-5.458) | **0.012*** |
| * = significant at 5% level. Nagelkerke r^2^ = 0.123. | | | | | |

*Supplementary Figure 3.0*: Accompanying ROC curve for major morbidity.


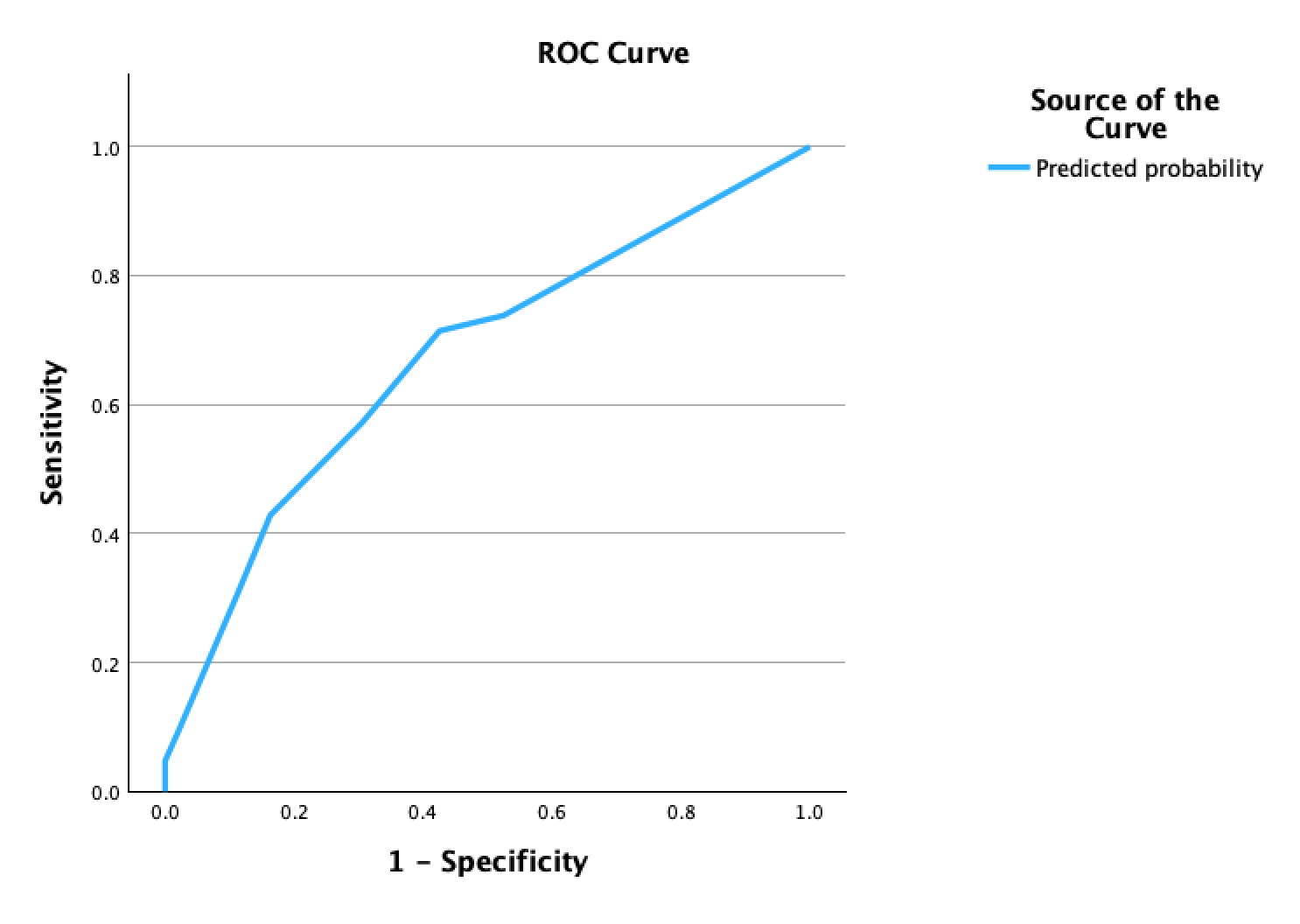


Area under the ROC curve = 0.672.

**Directed acyclic graphs**

All DAGs made using Dagitty.net ^(32)^.

*Supplementary Figure 4.0:* A DAG between SMI and major in-hospital morbidity.


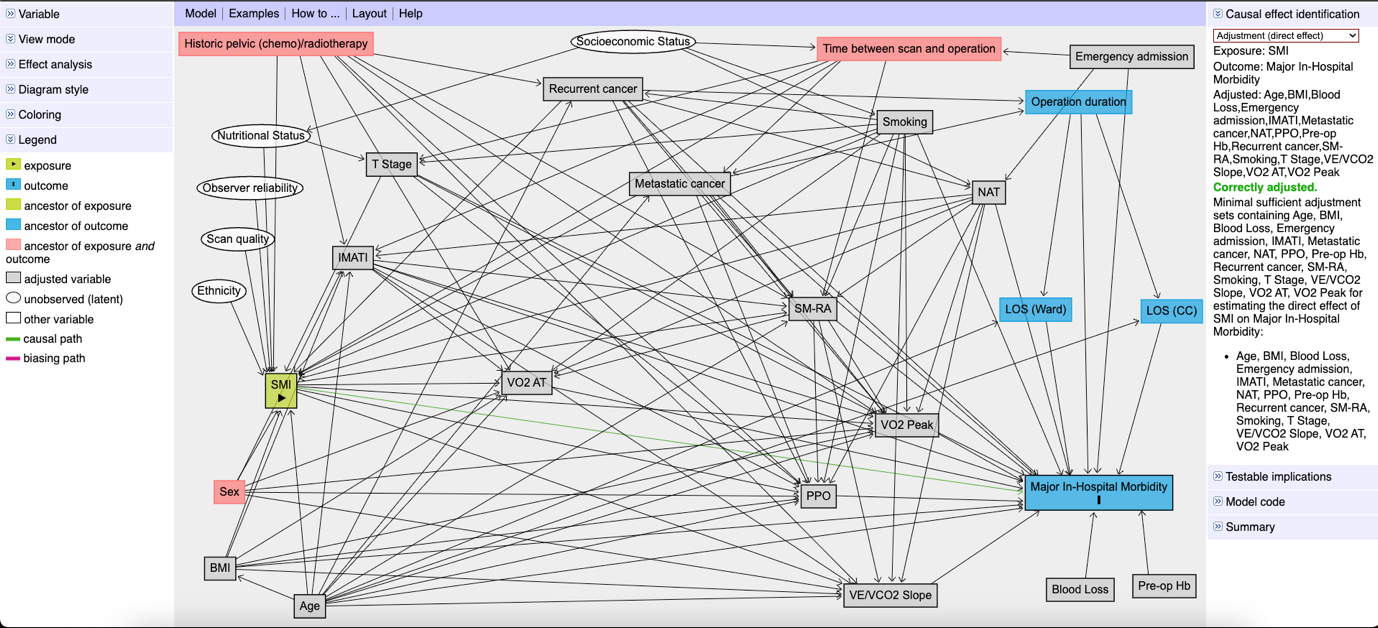


*Supplementary Figure 4.1:* A DAG between SM-RA and major in-hospital morbidity.


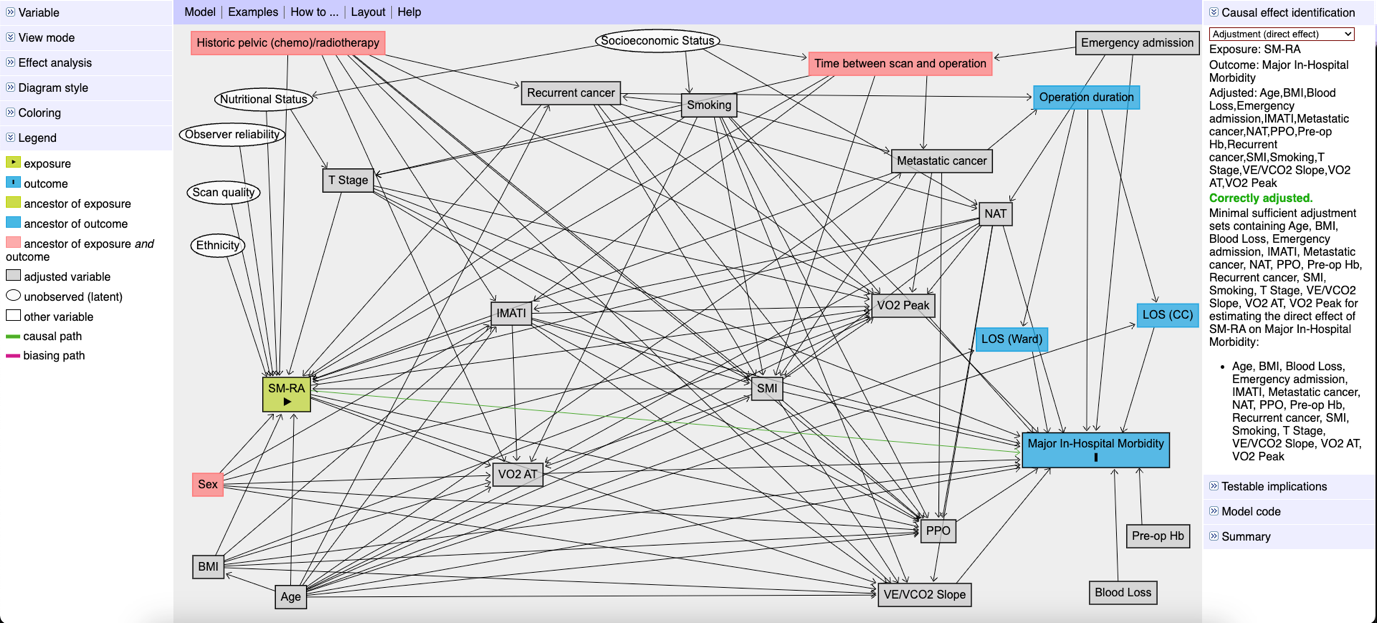


*Supplementary Figure 4.2:* A DAG between IMATI and major in-hospital morbidity.


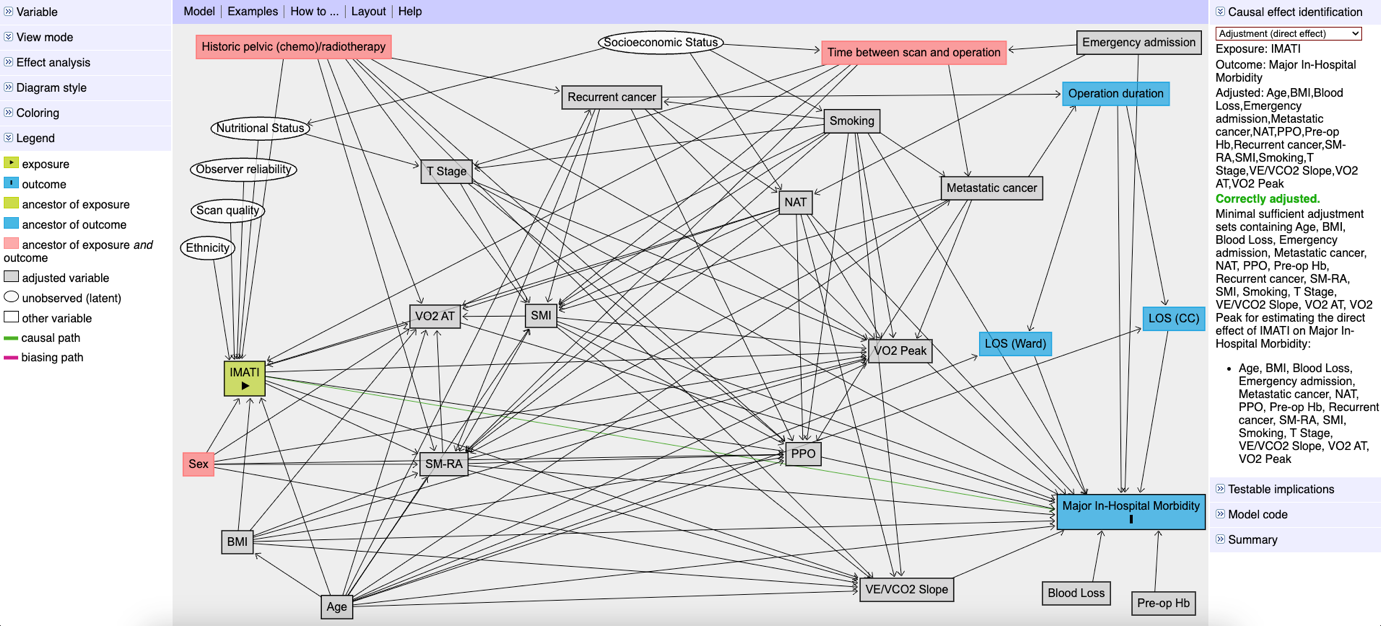


*Supplementary Figure 4.3:* A DAG between VO_2_ peak and major in-hospital morbidity.


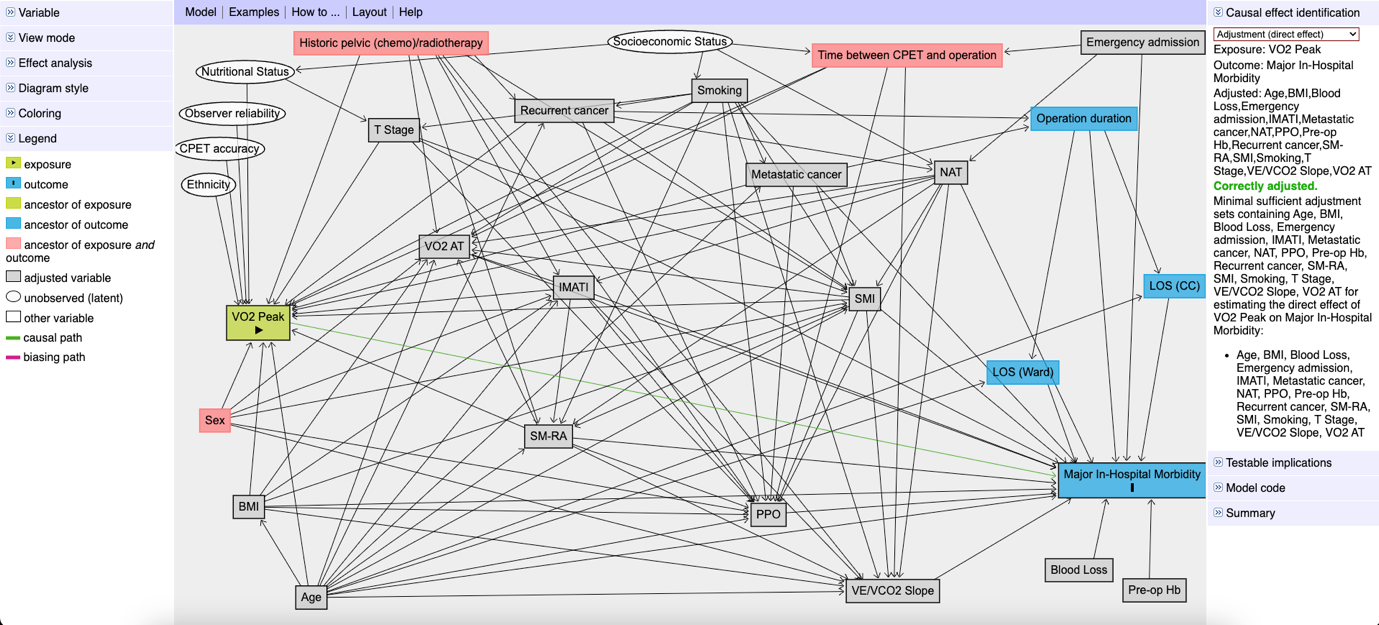


*Supplementary Figure 4.4:* A DAG between VO_2_ at AT and major in-hospital morbidity.


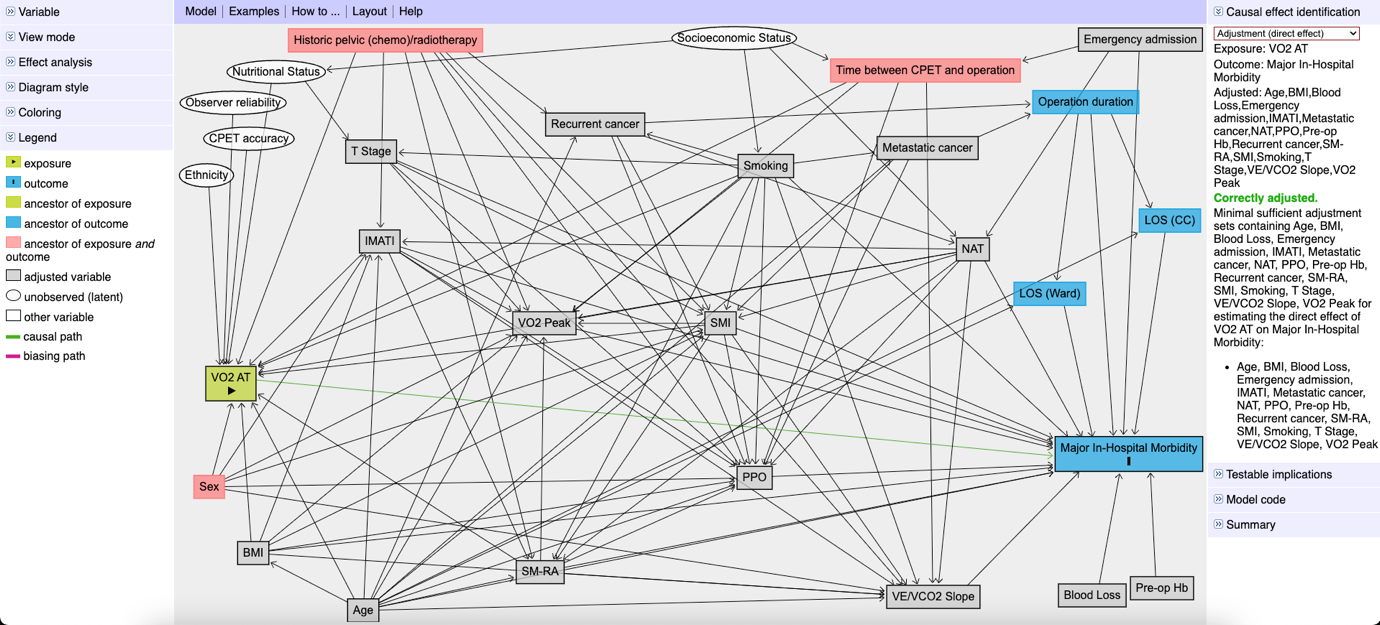


*Supplementary Figure 4.5:* A DAG between V*_E_*/VCO_2_ and major in-hospital morbidity.


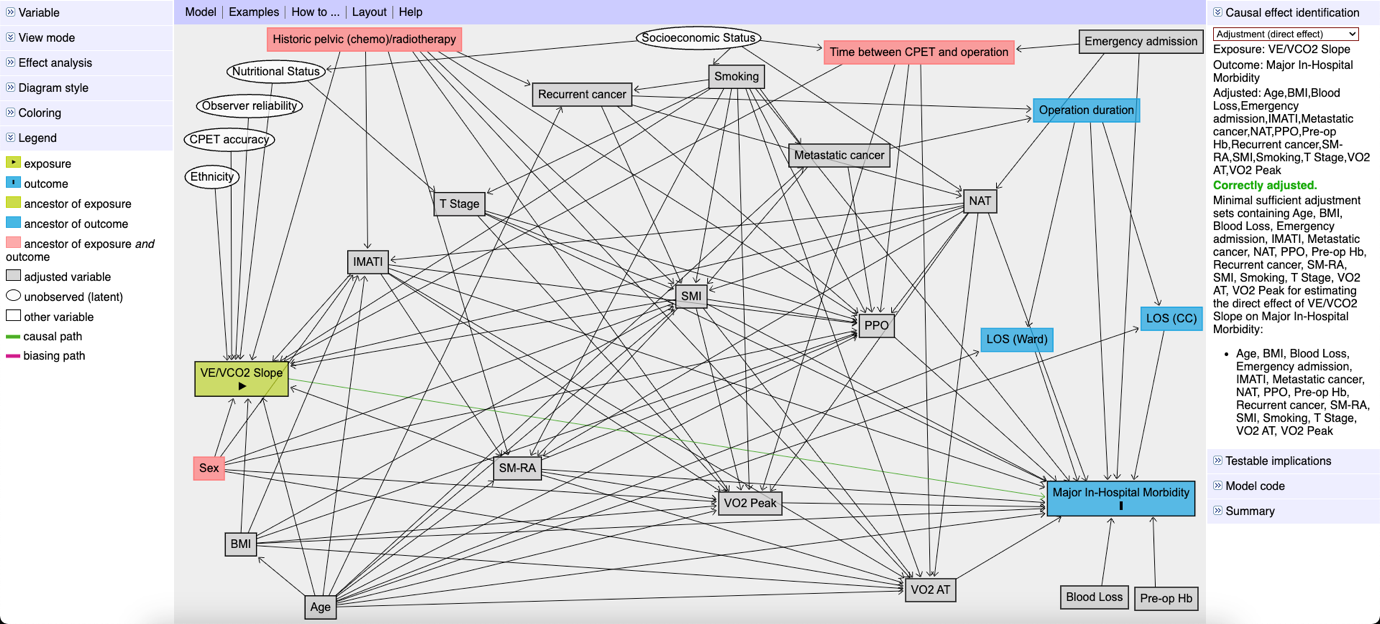


*Supplementary Figure 4.6:* A DAG between PPO and major in-hospital morbidity.


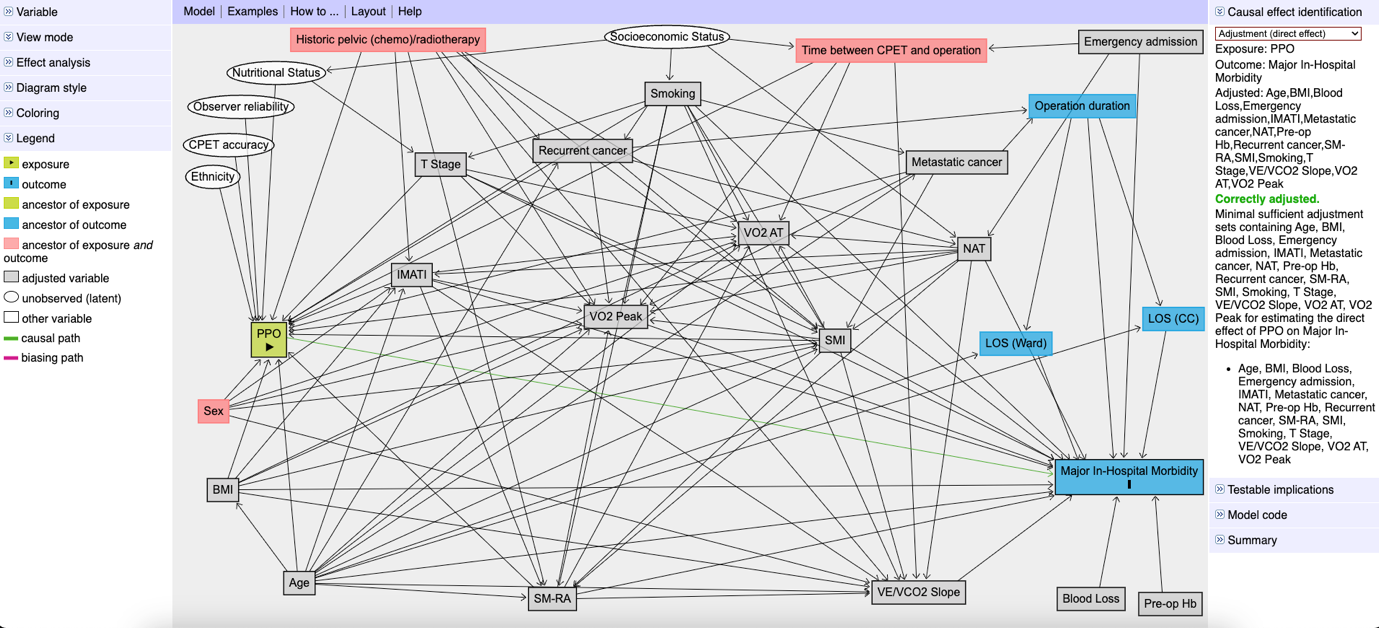

Supplement: Supplementary file 1 — Data S1: [file CODI-27-0-s001.docx]
